# The "Princess Christian" Hospital

**Published:** 1900-03-24

**Authors:** 


### THE "PRINCESS CHRISTIAN" HOSPITAL.

This hospital for the treatment of our troops in South Africa has been presented to the Government by Mr. Alfred Moseley, of West Lodge, Hadley Wood, Middlesex. This munificent gift has been entirely equipped and will be supported by the generous donor, who accompanies it to the Cape. Mr. Moseley's knowledge of the country and climate, gained during twenty-five years' connection with South Africa, will prove of considerable value to its efficient working. No trouble or expense has been spared to make the hospital in every way worthy of the patronage of her Royal Highness Princess Christian, who has graciously consented for it to bear her name. Major H. B. Mathias, D.S.O., R.A.M. Corps, who is a brother of the gallant Colonel H. H. Mathias, V.C., &c., of Dargai fame, goes out as Government representative with the hospital.

The civil staff will consist of the following: Chief surgeon—J. Paul Bush, M.R.C.S.Eng., Surgeon to the Bristol Royal Infirmary, Lecturer on Operative Surgery, University College, Bristol; chief surgeon Bristol Constabulary. Medical officers—G. V. Worthington, M.A., M.B., B.C.Cantab., M.R.C.S.Eng., late senior house surgeon St. Bartholomew's Hospital, London; late Og. district medical and sanitary officer, S. Canara, India. E. A. Nathan, M.D., B.S.London, M.R.C.S.Eng., late house surgeon and house physician St. Mary's Hospital, London; A. T. Fleming, M.R.C.S.Eng., late resident surgical officer Bristol Royal Infirmary. Dressers—A. B. Cridland, M.R.C.S.Eng., house surgeon Bristol Royal Infirmary; late casualty officer Bristol Royal Infirmary; E. M. Pearse, M.R.C.S.Eng., casualty officer Bristol Royal Infirmary. There will also be six non-commissioned officers and 26 hospital orderlies.

The building will consist of four pavilions, each 130 feet long and containing 25 beds—100 in all—with surgery, operation-room, nurses' room, fitted bath-rooms, washing-room for the men, and lavatories attached. The structure itself will be of corrugated iron, hung inside with greenish-tinted canvas. The wards will be comfortably furnished with bedsteads fitted with spring mattresses of the newest design, folding wash-stands, invalid tables and chairs, and every little detail likely to make them more comfortable and home-like. The surgeries and operation room will be fitted with all the latest improvements, including a very complete Röntgen ray apparatus, with all the latest inventions for localising foreign bodies, &c. The accessories include the new mercurial interruptor for making and breaking the current, and driven by a special motor of its own. This interruptor gives a particularly steady and distinct image with the fluorescent screen, and it also has the advantage of diminishing the length of time-exposure. An expert in this department is included in the list of the medical officers. In addition to the main buildings there will be three stores for the warehousing of the ample supply of provisions, invalid specialities, stimulants, drugs, &c. There will also be a separate building for the storage of the linen, a laundry, and a very complete kitchen of considerable size. In charge of the culinary department will be a professed cook and assistants. Another large structure will contain a central mess room and sleeping accommodation for the doctors, nurses, and other members of the staff, which will number in all about fifty persons. The plans and specifications of the hospital were prepared by Mr. Fred. W. Marks, A.R.I.B.A., of 3, Staple Inn, W.C., and the work has been admirably carried out under his superintendence by Messrs. Humphreys (Limited), of Knightsbridge.

